# Greenness and Birth Outcomes in a Range of Pennsylvania Communities

**DOI:** 10.3390/ijerph13030311

**Published:** 2016-03-11

**Authors:** Joan A. Casey, Peter James, Kara E. Rudolph, Chih-Da Wu, Brian S. Schwartz

**Affiliations:** 1 Robert Wood Johnson Foundation Health and Society Scholars Program, UC San Francisco and UC Berkeley, CA 94704, USA; Kara.Rudolph@ucsf.edu; 2 Departments of Epidemiology and Environmental Health, Harvard TH Chan School of Public Health, Boston, MA 02215, USA; pjames@hsph.harvard.edu; 3 Department of Forestry and Natural Resources, National Chiayi University, Chiayi City 60004, Taiwan; wu@hsph.harvard.edu; 4 Departments of Environmental Health Sciences and Epidemiology, Johns Hopkins Bloomberg School of Public Health, Baltimore, MD 21205, USA; bschwar1@jhu.edu; 5 Department of Medicine, Johns Hopkins School of Medicine, Baltimore, MD 21205, USA; 6 Center for Health Research, Geisinger Health System, Danville, PA 17822, USA

**Keywords:** greenness, pregnancy outcome, preterm birth, low birth weight, small for gestational age, propensity score, machine learning

## Abstract

Living in communities with more vegetation during pregnancy has been associated with higher birth weights, but fewer studies have evaluated other birth outcomes, and only one has been conducted in the Eastern United States, in regions with a broad range, including high levels, of greenness. We evaluated associations between prenatal residential greenness and birth outcomes (term birth weight, small for gestational age, preterm birth, and low 5 min Apgar score) across a range of community types using electronic health record data from 2006–2013 from the Geisinger Health System in Pennsylvania. We assigned greenness based on mother’s geocoded address using the normalized difference vegetation index from satellite imagery. We used propensity scores to restrict the study population to comparable groups among those living in green *vs.* less-green areas. Analyses were adjusted for demographic, clinical, and environmental covariates, and stratified by community type (city, borough, and township). In cities, higher greenness (tertiles 2–3 *vs.* 1) was protective for both preterm (OR = 0.78, 95% CI: 0.61–0.99) and small for gestational age birth (OR = 0.73, 95% CI: 0.58–0.97), but not birth weight or Apgar score. We did not observe associations between greenness and birth outcomes in adjusted models in boroughs or townships. These results add to the evidence that greener cities might be healthier cities.

## 1. Introduction

The biologist E.O. Wilson developed the theory of biophilia, which postulated that, since human beings evolved out of nature, they have a biologically-ingrained affinity for nature [1]. Indeed, studies have demonstrated that exposure to areas with higher amounts of community vegetation has many benefits, as would be expected by these evolutionary links, including higher levels of physical activity, lower levels of obesity, improved mental health outcomes, and lower cardiovascular disease and overall mortality [2]. The relationship between community vegetation and birth outcomes has been explored in Europe [3,4,5,6,7,8], Israel [9], Canada [10], and the U.S. [11,12,13]. These studies derived greenness—the density of vegetation in a given area—from satellite imagery as the normalized difference vegetation index (NDVI), which can range from −1.0 to 1.0 (higher values indicating higher greenness). This body of literature shows consistent evidence that living in areas with higher greenness during pregnancy is associated with higher birth weights (e.g., in Vancouver, British Columbia, a 0.1 increase in NDVI was associated with a 21 g increase in birth weight) [10]. However, findings varied for other birth outcomes, including preterm and small for gestational age birth [4,8,9,13].

The majority of studies on greenness and birth outcomes have been conducted in urban areas where absolute levels of greenness are low (NDVI < 0.4) and are often limited to within-city comparisons in small geographic areas. To our knowledge, only one other study has examined the relationship between greenness and birth outcomes across a range of communities, encompassing the full spectrum of rural to urban areas in a large geography in the United States [13]. Associations may not be consistent across communities that vary on individual (e.g., race/ethnicity, diet, education level), social, healthcare, environmental, and community factors. The relevant geographic context for greenness exposure may also differ by community. Such factors could result in community-based differences in observed relationships between environmental exposures and health [14,15,16,17]. For example, a recent study reported a significant association between greenness and reduced blood pressure among children in Munich, but no association among children in a more rural area [15].

We sought to evaluate the relationship between prenatal exposure to greenness and birth outcomes in a range of Pennsylvania communities. Such analysis, which included participants from diverse communities, while adjusting for individual and neighborhood-level confounders, may lead to practical violations of the positivity assumption (*i.e.*, participant subgroups in green areas with few or no comparable counterparts in less-green areas and *vice versa*). We addressed concerns about violations of the positivity assumption (*i.e.*, each individual has a non-zero probability of being in either treatment group) by (1) performing analyses separately by community type; and (2) using propensity scores derived from machine learning [18,19] to restrict our analysis to exchangeable groups. We were also able to account for potentially important factors not considered in prior studies, including exposure to animal feeding operations (AFOs) [20,21], unconventional natural gas development (UNGD) [22,23], and well water use [24].

## 2. Materials and Methods

### 2.1. Conceptual Framework

We proposed a conceptual framework outlining the mechanisms by which greenness might affect birth outcomes (Figure S1): by altering maternal levels of physical activity, reducing maternal stress, enhancing social contacts among mothers, reducing maternal noise and air pollution exposure, and moderating ambient temperatures [2,4]; and by which spatially-correlated environmental and social exposures and individual-level factors might confound or mediate this relationship. Although several exposures, including individual socioeconomic status (SES), UNGD, and AFOs likely have bi-directional associations with NDVI (*i.e.*, wealthier individuals may plant more trees, but greener neighborhoods might also attract individuals with more money), we chose to treat them as confounders in our analysis as we considered this the primary direction. We used the conceptual framework to guide our covariate selection, model building, and interpretation of results. 

### 2.2. Setting and Study Population

We used electronic health record (EHR) data on mothers who delivered neonates at one of two labor and delivery-equipped Geisinger Health System hospitals between 1 January 2006 and 31 January 2013. Geisinger’s primary catchment area covers nearly 40 counties, containing a range of self-governed Pennsylvania communities, including suburban and rural townships, moderate-to high-density boroughs, and cities, with median population densities ranging from 34 to 738 to 1360 people per km^2^, respectively. The Geisinger primary care population is representative of the general population of the region [22]. The Institutional Review Board at the Geisinger Health System approved this study (#00004966).

We identified mothers who gave birth and infants born at Geisinger from primary and non-primary care records using *International Classification of Diseases, Ninth Revision* codes V27.x and V30.x. We linked these mothers to their neonates in the EHR, primarily using medical record numbers, but also with addresses, names, and dates (*n* = 20,569 delivery events and *n* = 20,598 neonates). We geocoded the mother’s most recent address using ArcGIS 10.2 (Environmental Systems Research Institute, Redlands, CA, USA) with a series of basemaps [25]. We then assigned each home address to one of three community types, defined using U.S. Census minor civil division and census tract boundaries, which included 390 townships, 237 boroughs, and 124 census tracts in cities [26]. This mixed definition of place is thought to appropriately capture the cultural and geographic space significant to residents [26]. We excluded mothers and neonates who we could not match (*n* = 622 neonates, *n* = 433 deliveries), with primary addresses outside Pennsylvania (*n* = 492), those we could not geocode (*n* = 1389), non-singleton births (*n* = 1019), stillbirths (*n* = 13), and neonates with serious birth defects (*n* = 74), birth weights <500 g (*n* = 22), or gestational ages <22 weeks (*n* = 8) [22].

### 2.3. Outcome Assessment

We identified four birth outcomes: term birth weight (≥37 weeks), small for gestational age birth, preterm birth (<37 weeks gestation), and low 5 min Apgar score (<7). We used physician-estimated gestational age, which was determined based on mother-reported last menstrual period and 20 week ultrasound. We extracted birth weight and 5 min Apgar score from the EHR vitals file and labor and delivery notes (free text), and a separate labor and delivery database maintained continuously by nursing personnel. Small for gestational age was assigned based on an internal reference of babies born at Geisinger between 2006–2013 [27]. Neonates with a birth weight below the sex-specific 10th percentile for gestational age were considered small for gestational age. We dichotomized 5 min Apgar score at a clinically meaningful cutpoint (<7) where infants are more likely to require respiratory support and are at elevated risk of mortality [28].

### 2.4. Individual- and Community-Level Covariates

We obtained demographic characteristics from the EHR. These included neonate sex, gestational age, season and year of birth; maternal age, race/ethnicity, primary care status (primary or non-primary), smoking status during pregnancy, pre-pregnancy body-mass index, parity, number of antibiotic orders during pregnancy, and receipt of Medical Assistance, a means-tested program often used as a surrogate for low family socioeconomic status (SES) [25,29]. Smoking status was assigned from the social history, problem list, and medications files. If a mother smoked during gestation she was defined as an ever-smoker. We considered mothers to receive Medical Assistance when we identified receipt (from 1 of 24 codes) at >2 encounters [30]. Season of birth was assigned as winter (December–February), spring (March–May), summer (June–August), and fall (September–November) [31]. We used a mother’s home address to link potentially confounding spatial covariates: well water use [24]; community walkability [10,32]; community socioeconomic deprivation (CSD) [33,34]; distance to nearest road [35]; exposure to swine AFOs [20,21]; and UNGD [22,23], the latter three potential sources of air pollution. Due to sparse U.S. Environmental Protection Agency air quality monitors in the study area we were unable to include estimates of exposure to specific air pollutants. Distance to nearest major road, a surrogate for traffic-related air pollution [35], was calculated using 2013 U.S. Census Bureau Topologically Integrated Geographic Encoding and Referencing files. Swine AFOs may contribute to air pollution or allergen exposure [20]; we estimated exposure to swine AFOs using a cumulative metric that incorporated number of animals at each operation and the inverse-squared distance between operations and the home address [25]. To incorporate information about UNGD we used Pennsylvania Department of Environmental Protection (PADEP) and the Pennsylvania Department of Conservation and Natural Resources data to create a continuous variable of the number of unconventional wells drilled within 20 km of the home at the time of birth. Well water use was based on public water service areas compiled by the PADEP. We created a walkability metric that measured key features of urban development by z-transforming and summing three census block group level metrics: population density, land-use mix, and street network connectivity, using data compiled by the U.S. Environmental Protection Agency through Smart Location Database [36]. CSD, a version of the Townsend Index modified for use in rural communities, was generated by z-transforming and summing six American Community Survey (2006–2010) minor civil division level variables: proportion of the population with less than a high school education, unemployed, not in the labor force, in poverty, receiving public assistance, and households without a car [37].

### 2.5. Greenness Exposure Assessment

**Figure 1:**
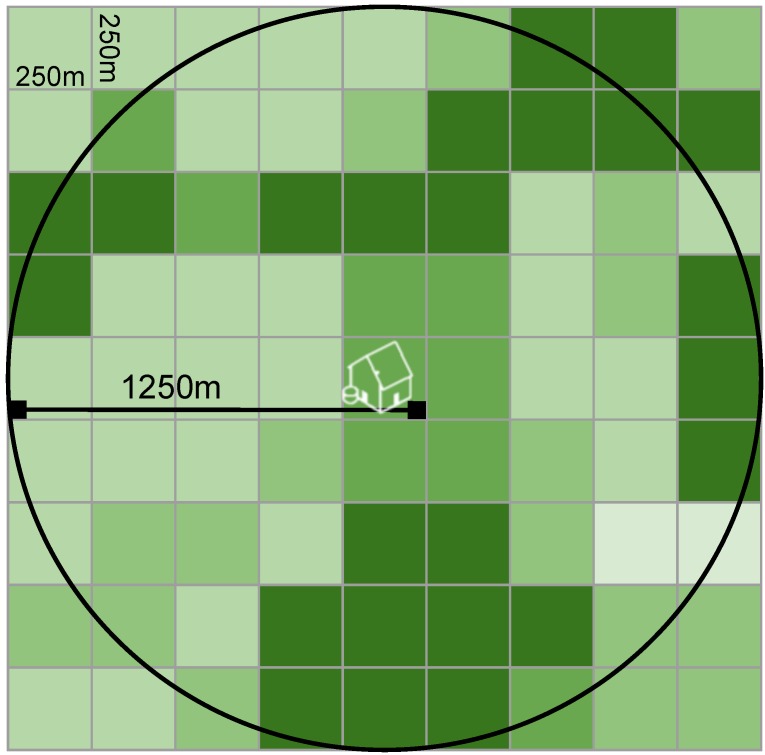
Schematic of residential greenness measurement, which was completed by taking the average of NDVI in a 5-pixel (1250 m) radius surrounding and including the home in the three seasons preceding birth.

Exposure to green, natural areas around each home address was estimated using NDVI, a satellite-image based vegetation index. Chlorophyll in plants absorbs visible light for use in photosynthesis, while leaves reflect near-infrared light. NDVI calculates the ratio of the difference between the near-infrared region and red reflectance to the sum of these two measures, and ranges from −1.0 to 1.0, with larger values indicating higher levels of vegetative density [38,39,40]. For this study, we used data from the Moderate-resolution Imaging Spectroradiometer (MODIS) from the National Aeronautics and Space Administration’s Terra satellite. MODIS provides composite images at a 250 m resolution every 16 days over the study period. We linked NDVI imagery to geocoded addresses and calculated the average greenness in the 250 m and the 1250 m radius including and surrounding the home address (Figure 1). We chose the 250 m radius as a measure of greenness directly accessible outside each home, and the 1250 m radius as a measure of greenness within a 10–15 min walk based on prior work on neighborhood environments and health behaviors [41]. We assigned each mother the average greenness at her home address based on greenness data in the 3 seasons prior to her child’s birth date, unless the child was born more than halfway through a season, in which case the greenness in the two prior seasons and the season of birth were assigned.

### 2.6. Statistical Analysis

**Figure 2:**
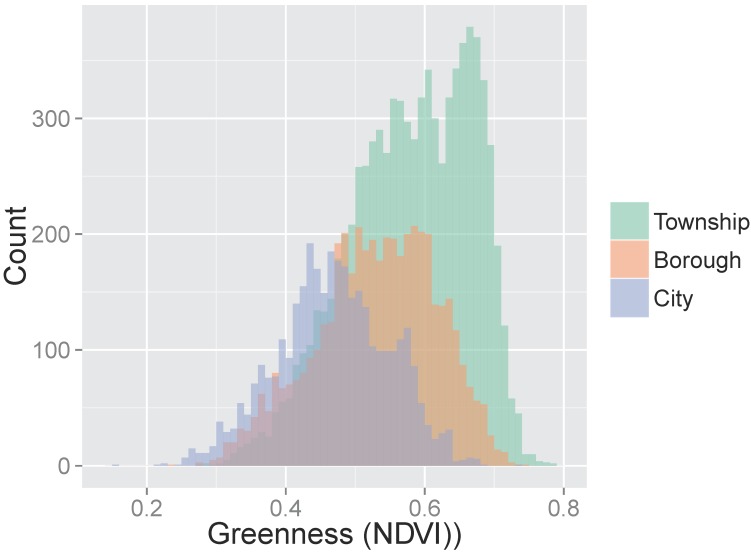
Distribution of greenness (NDVI) by community type.

The distribution of greenness differed by community type (Figure 2). We were concerned about lack of overlap in distributions of variables strongly correlated with both NDVI and birth outcomes among women living in more *vs.* less green communities (e.g., low proportion of black mothers in townships where levels of greenness were highest). This could result in model extrapolation rather than adjustment in evaluating our primary associations of interest. We also hypothesized that the association between greenness and birth outcomes might operate differently in different community types. To make the compared groups more similar on greenness and covariates, we first stratified our analyses by community type (*i.e.*, city, borough, and township) and then confined analysis to comparable patients. 

To restrict our analysis to comparable mothers we evaluated the predicted probabilities of greenness as a function of baseline covariates including maternal age, race/ethnicity, alcohol and tobacco use, number of prenatal clinical visits, receipt of Medical Assistance, and UNGD exposure, and others. We compared the distributions of the predicted probabilities (*i.e.*, propensity scores [19]) of living in a more green area (tertiles 2–3 of greenness) as a function of baseline covariates, within each community type. Propensity scores were estimated using super learning [42], an ensemble machine learning algorithm that has been used previously to improve propensity score estimation [43,44] The Super Learner package optimally weights multiple machine learning algorithms (e.g., generalized additive models, neural networks) to minimize the V-fold cross-validated prediction error. In predicting a complex exposure like exposure to residential greenness, using machine learning may present an advantage in reducing reliance on correct specification of the parametric exposure model. 

**Figure 3:**
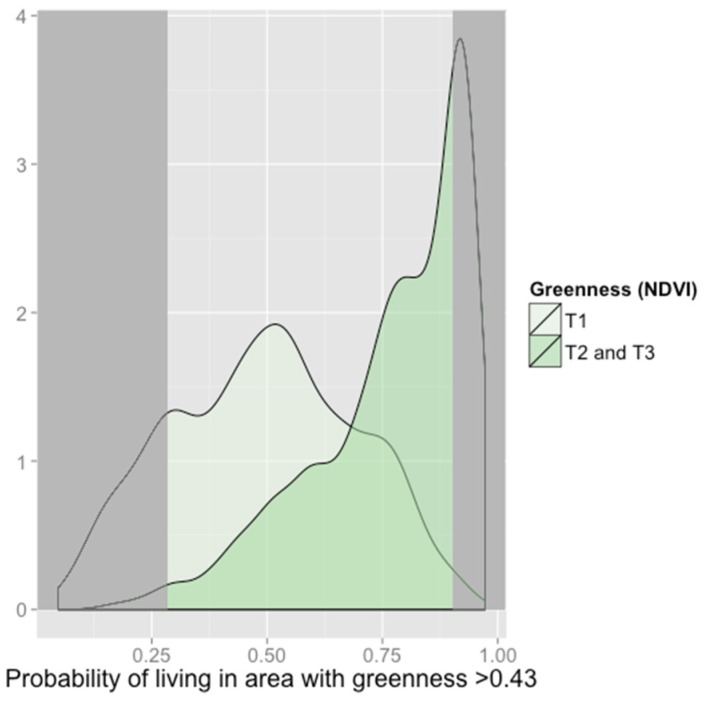
Propensity score distributions for patients in cities comparing those with NDVI values in the 2nd and 3rd tertiles (darker green) to the 1st (lighter green). The propensity score is the predicted probability of living in tertiles 2 or 3 (NDVI > 0.43), given baseline covariates. The analysis was restricted to participants with propensity scores outside of dark grey shaded regions to ensure that those living in green and non-green environments were comparable.

Plots confirmed different distributions of predicted probabilities of high greenness exposure (Figure 3 (cities) and Figure S2 (cities, boroughs, and townships)). As has been recommended in order to reduce model extrapolation [45], we restricted the population, by community type, to those with propensity scores between the 1st propensity score percentile among those living in green environments (tertiles 2 and 3) and the 99th propensity score percentile among those living in non-green environments (tertile 1). For example, in cities, for 29% of participants whose NDVI values were in the 2nd or 3rd tertile, there were no comparable participants with NDVI values in the 1st tertile; likewise, for 18% of participants with NDVI values in the 1st tertile, there were no similar participants with NDVI values in the 2nd and 3rd tertile. Restricting to this area of overlap in the distributions ensured that each participant had a counterpart comparable on propensity to live in a greener environment.

We evaluated associations between residential greenness (tertile 2–3 *vs.* tertile 1) and four birth outcomes using linear and logistic regression, depending on outcome, accounting non-independent observations due to neonates nested within mothers (*i.e.*, multiple singleton births per mother) and mothers nested within communities in our estimated standard errors [46]. Adjusted models controlled for neonate sex, year and season of birth, maternal age at delivery (linear and quadratic, years), maternal race/ethnicity (white, black, Hispanic), primary care status (yes *vs.* no), smoking status during pregnancy (never *vs.* ever), pre-pregnancy body mass index (underweight: <18.5 kg/m^2^; normal: 18.5–24.9 kg/m^2^; overweight: 25–29.9 kg/m^2^; or obese: ≥30 kg/m^2^), parity (nulliparous *vs.* multiparous), receipt of Medical Assistance (never *vs.* ever, surrogate for low family SES), number of antibiotic orders during pregnancy, distance to nearest major road (meters), drinking water source (municipal *vs.* non-municipal), the number of unconventional natural gas wells drilled prior to birth within 20 km of the home, exposure to swine operations, block group walkability (continuous), and CSD (quartiles). We tested all two-way interactions between model covariates and retained those that significantly improved model fit. We also specifically evaluated whether CSD or receipt of Medical Assistance modified relations between residential greenness and birth outcomes by including interaction terms. We used quadratic and cubic terms to assess non-linear relationships.

Models were selected using *a priori* hypotheses about confounders and likelihood ratio testing (*p* < 0.05). Residual semivariograms showed that model residuals did not exhibit spatial autocorrelation. In the original population data missingness was minimal (0%–5.8% on outcomes and 0%–5.2% on confounders) and in the restricted population data was not missing on confounders and again minimal for outcomes (0%–5.0%), thus we excluded mothers from models when they were missing data. Statistical analyses were implemented using Stata version 13 (StataCorp., College Station, TX, USA) and R version 3.1.1 (R Core Team, Vienna, Austria) and the R SuperLearner package [42].

### 2.7. Sensitivity Analyses

To evaluate the sensitivity of associations to choices about NDVI metric and residential environment size, we calculated greenness exposure based on mean and maximum greenness during pregnancy in 250 m and 1250 m radii. To test sensitivity to the greenness cutpoint we also evaluated associations dichotomizing greenness at the community-specific 20th-percentile (comparing <20th-percentile to ≥20th-percentile). Finally, we evaluated the association between continuous NDVI and birth outcomes in both the original and restricted population.

## 3. Results

**Figure 4:**
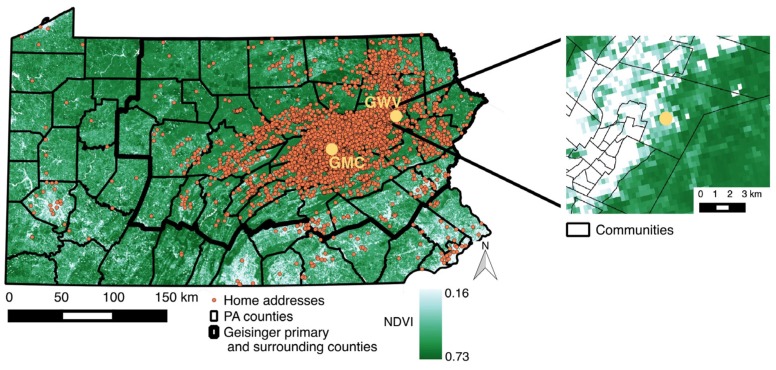
Distribution of greenness (normalized difference vegetation index (NDVI)) statewide overlaid with the location of Geisinger Medical Center (GMC) and Geisinger Wyoming Valley (GWV), the delivery hospitals, and most recent home addresses of mothers who delivered at the Geisinger Health System, Pennsylvania (PA), 2006–2013.

From January 2006 through January 2013, a total of 16,184 mothers delivered 20,598 infants at two Geisinger Health System hospitals. After exclusion, we had a sample size of 16,913 mother-infant pairs. The use of propensity scores to further restrict to exchangeable groups left 12,821 infants (2563 in cities, 3797 in boroughs, and 6461 in townships) born to 10,787 mothers (Figure 4). The median (interquartile range) term birth weight in cities, boroughs, and townships was 3308 g (3010 g–3628 g), 3360 g (3068 g–3668 g), and 3390 g (3090 g–3697 g), respectively. More infants were born preterm and small for gestational age in cities (13% and 13%) compared to boroughs (11% and 10%) and townships (11% and 9%). The overall prevalence of a low 5 min Apgar score was low (2%). The proportion of mothers who resided in cities and lived in the top 66.7% (2nd or 3rd tertile) of the overall NDVI distribution was just 29% (Table 1). Season of birth was strongly associated with NDVI tertile with neonates born in spring much more likely to fall in 2nd or 3rd tertile across community types. Women living in cities were more likely to receive Medical Assistance than women in boroughs and townships, and within cities women residing in areas with lower greenness were more likely to receive Medical Assistance; several other covariates also differed between community types, as well as within community by NDVI tertile.

We did not observe significant associations between residential greenness and term birth weight or 5 min Apgar score in any community type (Table 2), though birth weight associations in cities were suggestive of a protective effect. In adjusted analyses, higher NDVI was associated with lower odds of small for gestational age births in cities, but not in boroughs or townships. Higher levels of residential greenness in cities were also associated with lower odds of preterm birth, an association that was not apparent in the original, non-restricted population that included non-comparable participants (Table S1). We did not observe evidence of effect modification by Medical Assistance or CSD (results not shown).

In a sensitivity analysis, we observed high correlation (Spearman > 0.7) between mean and maximum residential greenness defined by 250 m and 1250 m radii. Analysis produced similar associations for both buffer sizes (results not shown). In evaluation of sensitivity of results to decisions regarding NDVI cutpoints, a division at the 20th percentile yielded similar associations to the primary analysis (results not shown). Analysis in the restricted population with a continuous NDVI variable suggested a statistically significant relationship between continuous NDVI and preterm birth in cities (Table S2). Additional greenness also appeared protective of reduced term birth weight in cities, but the association was not statistically significant. Continuous NDVI was not associated with birth outcomes in boroughs or townships and there did not appear to be a departure from linearity in any community as assessed by quadratic and cubic terms and splines.

## 4. Discussion

In an analysis of birth outcomes across a range of communities, from rural to urban, in Pennsylvania, we found that higher levels of residential greenness, in cities only, were associated with lower odds of preterm and small for gestational age births. The analysis presented challenges, for reasons often not recognized or acknowledged in environmental epidemiologic studies. Specifically, we had to address structural confounding by restricting the analysis to mothers with comparable counterparts while controlling for numerous spatially correlated exposures. Prior studies of greenness in cities did not have to deal with this methodological complication. As studies of contextual and community influences on health increase in their geographic scope, they will continue to run into complex data problems including issues of structural confounding. We provide an example of how to use machine-learning based propensity scores and stratification to study communities ranging from urban to very rural while reducing the possibility that the associations were explained by either model extrapolation or confounding due to a number of important environmental exposures and conditions.

In addition to the methodological contributions we also add data from outside cities to the greenness and birth outcome literature. In our study, greenness only conferred benefits in cities. This could suggest that in communities with higher absolute levels of greenness, for example boroughs and townships, additional greenness does not benefit neonate health.

Greenness exposure may provide opportunities for physical activity, lower psychophysiological stress and depression, increase social interactions, and lower exposure to noise, air pollution and extreme temperatures (Figure S1). These potential mediators have been linked to birth outcomes, including birth weight, intrauterine growth, gestational age at delivery, and maternal mental health [47,48,49,50,51,52], which may explain our observed association between increased greenness and lower odds of SGA and preterm birth. High ambient temperatures have been linked to adverse pregnancy outcomes [53], and greenness may buffer heat islands within urban areas [54]. This may partially explain why we observed associations within participants who lived in cities, but not in rural areas where heat island effects were unlikely to occur.

Two prior studies reported greenness as protective of preterm birth. In Southern California, Laurent *et al.* [12] found that an IQR increase in NDVI (0.13) in the 150 m area around each mother’s home was associated with 2% lower odds of preterm birth in models adjusted for air pollution and traffic density (*p* = 0.05). An analysis of 64,705 singleton births in Vancouver, Canada showed that a 0.1 increase in NDVI was associated with a 5% decrease (95% CI: 1%, 9%) in the odds of a moderately preterm (30–36 weeks) birth [10], adjusting for air pollution, noise, and neighborhood walkability. 

Three studies have also reported that greenness is protective for small for gestational age birth. The Vancouver study found that a 0.1 increase in NDVI was associated with a 3% (95% CI: 0%–6%) decrease in small for gestational age birth [10]. Donovan *et al.* [11] found that a 10% increase in tree canopy cover in the 50 m surrounding a mother’s home in Portland, Oregon was associated with 1.42 fewer small for gestational age babies per 1000 births. A recent study in Connecticut reported that an IQR increase in NDVI (0.38) was associated with 2.6% fewer (95% CI: −5.1–−0.1) small for gestational age births [13].

Most studies of greenness and birth outcomes have reported that NDVI is associated with continuous birth weight [4,5,6,8,9,10,12]. We found that neonates in cities in the 2nd and 3rd tertile of greenness weighed 42 g (95% CI: −1, 85) more than those in the 1st tertile and that a 0.1 unit increase in NDVI in cities was associated with an increase in term birth weight of 21 g (95% CI: −7, 49), although neither association crossed an inferential boundary. Hystad *et al.* (2014) observed a similar effect size, 20.6 g (95% CI 16.5, 24.7) per 0.1 unit increase in NDVI. Our findings were also similar in magnitude compared to those seen in other studies [4,6,8,9], but larger than those observed by Laurent *et al.* and Ebisu *et al.* in the U.S. [12,13]. Differences may result from different distributions of socioeconomic or other factors or different absolute levels of greenness among study populations, which may affect susceptibility to greenness exposures and birth weight. Additionally, we restricted our study population to comparable groups. While we were able to account for unconventional natural gas development and animal feeding operations, other studies were able to include factors such as noise or maternal education in their analyses, and these model specifications could have led to different estimated associations. 

Prior studies have reported stronger associations of greenness with birth outcomes among individuals with low SES or living in poorer communities [4,5,6,9]. We did not find evidence of effect modification by family SES (*i.e.*, Medical Assistance) or CSD. Since more mothers on Medical Assistance resided in cities and cities in general had higher levels of community deprivation, it is possible that we did not observe effect modification by family SES and community deprivation since we stratified analyses by community type.

Our study had limitations. First, while stratifying our analysis by community type and restricting to comparable populations to prevent model extrapolation was a key strength, this methodology prevented us from testing interactions between community type and greenness, or greenness and overall CSD or modeling NDVI as a continuous variable in our main analysis. We cautiously present linear associations between continuous NDVI and birth outcomes in the Supplemental Appendix (Table S2). Second, we had some misclassification of exposure and other covariates since the EHR only maintains each mother’s most recent address. However, previous analysis in this population suggests residential stability is high. We compared the addresses of >300,000 Geisinger patients and determined that 86% did not change address or moved <1500 m between 2010–2013 [22]. Third, maternal SES is a known risk factor for poor birth outcomes [55,56]. It is likely we did not fully adjust for this potential confounder since we used receipt of Medical Assistance as a proxy for family SES because the EHR data did not include information on mothers’ occupation, wealth, or education. Fourth, the EHR also lacked other relevant data on maternal nutrition and exercise. Fifth, we cannot describe qualitatively the types of vegetation associated with improved outcomes or how mothers interacted with their green space (e.g., increased physical activity, reduced stress) and therefore cannot know how living in a greener area may account for better birth outcomes. Sixth, we lacked direct measurement of noise and air pollution. As proxies for air pollution we used distance to nearest road and number of unconventional gas wells within 20 km of the mother’s residence. While not comprehensive, because air pollution is emitted from other industrial activities, these variables may be adequate in this large region with a range of community settings. Finally, we calculated NDVI around the participants’ homes, but did not have information about their workplaces or other natural environment exposures. In general, uncertainty in this area of study persists over the appropriate location and scale for accurately measuring environmental exposures. 

Despite these limitations, our analysis offered improvements over past studies. A main contribution is limiting analysis to the area of common support, which prevented reliance on model extrapolation. The EHR provided detailed patient data (e.g., antibiotic orders during pregnancy) and accurate outcome ascertainment. Our NDVI exposure summarized exposure across pregnancy by averaging NDVI by trimester. We were able to assign a suite of confounders including information on drinking water source, industrial agriculture, and unconventional gas wells not available in other analyses. Although it is possible that including some of these factors may have over-controlled for exposure, each was hypothesized to act primarily as a confounder and was included based on substantive knowledge. 

## 5. Conclusions

We observed an association between higher levels of greenness and lower odds of preterm and small for gestational age birth only among city-dwelling mothers. This finding was observed after restriction to mothers with similar probabilities of residing in areas with greenness and adjustment for a host of spatially correlated social and environmental exposures. Future environmental epidemiology studies that include participants from a range of communities should consider the issue of non-positivity in analyses. While more research is needed to identify the specific mechanisms through which greenness may impact birth outcomes, planners and policy makers should consider incorporating additional vegetation in urban areas.

## Figures and Tables

**Table 1:** Study population demographics restricted sample, *n* = 12,821.

Variable, *n* (%) unless Specified	City *n* = 2563		Borough *n* = 3797		Township *n* = 6461		
	Community-Specific NDVI Tertile		Community-Specific NDVI Tertile		Community-Specific NDVI Tertile		
	1, *n* = 927	2–3, *n* = 1636	1, *n* = 1253	2–3, *n* = 2544	1, *n* = 2249	2–3, *n* = 4212
NDVI, mean (SD)	0.39 (0.04)	0.50 (0.05)	0.44 (0.04)	0.57 (0.05)	0.48 (0.05)	0.62 (0.05)
NDVI, tertile 1 for all community types	927 (100)	889 (54.3)	1253 (100)	10 (< 1)	1195 (53.1)	0
NDVI, tertiles 2–3 for all community types	0	747 (45.7)	0	2534 (100)	1054 (46.9)	4212 (100)
**Maternal characteristics**						
Age, median (IQR)	28.3 (24.0–32.3)	28.6 (24.4–32.8)	26.9 (23.2–30.8)	26.8 (22.8–31.0)	25.1 (21.7–30.0)	25.6 (21.8–30.1)
Nulliparous	438 (47.2)	752 (46)	587 (46.8)	1141 (44.9)	1148 (51.0)	2024 (48.1)
Multiparous	489 (52.8)	884 (54)	666 (53.2)	1403 (55.1)	1101 (49.0)	2188 (51.9)
Race/ethnicity						
White	640 (69.0)	1231 (75.2)	1149 (91.7)	2359 (92.7)	2126 (94.5)	4037 (95.8)
Black	160 (17.3)	215 (13.1)	54 (4.3)	98 (3.9)	49 (2.2)	72 (1.7)
Hispanic	106 (11.4)	171 (10.5)	41 (3.3)	65 (2.6)	42 (1.9)	53 (1.3)
Other	21 (2.3)	19 (1.2)	9 (0.7)	22 (0.9)	32 (1.4)	50 (1.2)
Ever smoker	148 (16.0)	333 (20.4)	280 (22.4)	587 (23.1)	501 (22.3)	901 (21.4)
No receipt of medical assistance	356 (38.4)	690 (42.2)	645 (51.5)	1338 (52.6)	1435 (63.8)	2815 (66.8)
Receipt of medical assistance	571 (61.6)	946 (57.8)	608 (48.5)	1206 (47.4)	814 (36.2)	1397 (33.2)
Pre-pregnancy body mass index, kg/m^2^						
Normal (18.5–24.9)	386 (41.6)	740 (45.2)	523 (41.7)	1127 (44.3)	897 (39.9)	1715 (40.7)
Overweight (25.0–29.9)	247 (26.6)	360 (22.0)	328 (26.2)	634 (24.9)	616 (27.4)	1185 (28.1)
Obese (≥30.0)	275 (29.7)	495 (30.3)	375 (30.0)	728 (28.6)	694 (30.9)	1225 (29.1)
Underweight (<18.5)	19 (2.0)	41 (2.5)	27 (2.2)	55 (2.2)	42 (1.9)	87 (2.7)
No antibiotic orders during pregnancy	644 (69.5)	1195 (73.0)	925 (73.8)	1945 (76.5)	1700 (75.6)	3299 (78.3)
≥1 antibiotic orders during pregnancy	283 (30.5)	441 (27.0)	328 (26.2)	599 (23.5)	549 (24.4)	913 (21.7)
Non-primary care patient	593 (64.0)	1012 (61.9)	652 (52.0)	1289 (50.7)	1057 (47.0)	1973 (46.8)
Primary care patient	334 (36.0)	624 (38.1)	601 (48.0)	1255 (49.3)	1192 (53.0)	2239 (53.2)
Community socioeconomic deprivation, median (IQR)	4.4 (2.5–6.6)	4.1 (2.2–6.5)	0.6 (−1.0–2.5)	0.6 (−1.1–2.6)	−1.4 (−2.5–0)	−1.6 (−2.8–0)
Walkability z-score, median (IQR)	2.0 (1.1–3.3)	1.8 (0.5–3.1)	0.8 (−0.1–1.9)	0.7 (−0.2–1.8)	−1.2 (−1.9–−0.5)	−1.2 (−2.4–−0.9)
Distance to nearest major road (m), median (IQR)	278 (137–535)	309 (141–610)	411 (167–1034)	498 (175–1173)	1784 (611–5017)	2444 (735–6121)
Drilled unconventional natural gas wells within 20 km of home, mean (SD)	5 (21)	8 (26)	21 (89)	22 (86)	43 (131)	38 (118)
Swine operation exposure (animal units per km^2^ surrounding home), median (IQR)	5.9 (5.6–21.3)	6.3 (5.6–21)	10.8 (5.6–34.1)	11.2 (5.7–35.0)	16.6 (6.6–32.8)	16.3 (6.3–31.9)
**Infant characteristics**						
Female	450 (48.5)	817 (49.9)	617 (49.2)	1236 (48.6)	1099 (48.9)	2036 (48.3)
Male	477 (51.5)	819 (50.1)	636 (50.8)	1308 (51.4)	1150 (51.1)	2176 (51.7)
Year of birth						
2006–2007	209 (22.5)	343 (21.0)	262 (20.9)	686 (27.0)	410 (18.2)	1349 (32.0)
2008–2010	403 (43.5)	708 (43.3)	601 (48.0)	1092 (42.9)	1215 (54.0)	1867 (44.3)
2011–2013	315 (34.0)	585 (35.8)	390 (31.1)	766 (30.1)	624 (27.7)	996 (23.6)
Season of birth						
Winter (December–February)	277 (29.9)	366 (22.4)	341 (27.2)	683 (26.8)	520 (23.1)	1040 (24.7)
Spring (March–May)	49 (5.3)	589 (36.0)	78 (6.2)	851 (33.5)	124 (5.5)	1509 (35.8)
Summer (June–August)	237 (25.6)	420 (25.7)	318 (25.4)	654 (25.7)	555 (24.7)	1120 (26.7)
Fall (September–November)	364 (39.3)	261 (16.0)	516 (41.1)	356 (14.0)	1050 (46.7)	543 (12.9)
Term birth weight, median (IQR)	3293 (2993–3606)	3312 (3028–3637)	3345 (3060–3666)	3370 (3072–3668)	3390 (3090–3697)	3391 (3090–3698)
Small for gestational age birth	126 (13.6)	180 (11.0)	109 (8.7)	237 (9.3)	210 (9.3)	352 (8.4)
Preterm birth (<37 weeks gestational age)	127 (13.7)	195 (11.9)	117 (9.3)	297 (11.7)	248 (11.1)	465 (11.0)
5 min Apgar score <7	17 1.8)	41 (2.5)	26 (2.1)	39 (1.5)	42 (1.9)	88 (2.1)

**Table 2:** Unadjusted and adjusted associations of residential greenness and birth outcomes in restricted sample.

Variable ^b^	Unadjusted				Adjusted ^a^			
	Term Birth Weight (g)	Small for Gestational Age	Preterm Birth	5 min Apgar Score <7	Term Birth Weight (g)	Small for Gestational Age	Preterm Birth	5 min Apgar Score <7
	β (95% CI)	OR (95% CI)	OR (95% CI)	OR (95% CI)	β (95% CI)	OR (95% CI)	OR (95% CI)	OR (95% CI)
City, N births	2125	2420	2460	2497	2125	2420	2460	2497
Greenness T1	0	1	1	1	0	1	1	1
Greenness T2–3	36 (−5–78)	0.77 (0.60–1.00)	0.85 (0.67–1.07)	1.24 (0.82–1.87)	42 (−1–85)	0.73 (0.58–0.97)	0.78 (0.61–0.99)	1.42 (0.69–2.92)
Borough, N births	3184	3565	3609	3658	3184	3565	3609	3658
Greenness T1	0	1	1	1	0	1	1	1
Greenness T2-3	−3 (−28–21)	1.09 (0.90–1.32)	1.29 (1.02–1.62)	1.08 (0.75–1.53)	4 (−22–30)	1.19 (0.88–1.59)	1.25 (0.96–1.59)	0.94 (0.57–1.55)
**Township, N births**	5546	6205	6281	6209	5546	6205	6281	6209
Greenness T1	0	1	1	1	0	1	1	1
Greenness T2–3	5 (−18–29)	0.89 (0.73–1.09)	1.00 (0.85–1.18)	1.15 (0.81–1.65)	2 (−21–24)	0.90 (0.71–1.15)	0.95 (0.79–1.15)	1.20 (0.79–1.82)

**^a^** All models were adjusted for year and season of birth, sex, and gestational age of the neonate; maternal characteristics: age at delivery, race/ethnicity, primary care patient status, ever smoking, pre-pregnancy body mass index, parity, number of antibiotic orders during pregnancy, receipt of Medical Assistance, delivery hospital, drinking water source, distance to nearest major road, proximity to swine livestock operations, number of unconventional natural gas wells within 20 km; and community socioeconomic deprivation quartile and community walkability. The birth weight model was additionally adjusted for gestational age. Interactions that improved model fit were additionally included; **^b^** Greenness (NDVI) tertile cut points differed by community type: city = 0.43; borough = 0.49; and township = 0.54.

## Supplementary Materials

The following are available online at www.mdpi.com/1660-4601/13/3/311/s1, Table S1: Unadjusted and adjusted associations of tertiles 2–3 *versus* tertile 1 of residential greenness exposure and pregnancy outcomes in original population, Table S2: Unadjusted and adjusted associations of continuous residential greenness exposure and pregnancy outcomes in original and restricted populations, Figure S1: Conceptual framework with relations among residential greenness, confounders, mediators and birth outcomes, Figure S2: Propensity score distributions for patients in (**A**) boroughs; (**B**) townships; and (**C**) cities comparing those with NDVI values in the 2nd and 3rd tertiles.

## Abbreviations

The following abbreviations are used in this manuscript:

AFO	Animal feeding operation
CSD	community socioeconomic deprivation
EHR	electronic health record
GMC	Geisinger Medical Center
GWV	Geisinger Wyoming Valley
MODIS	Moderate-resolution Imaging Spectroradiometer
NDVI	normalized difference vegetation index
PADEP	Pennsylvania Department of Environmental Protection
SES	socioeconomic status
UNGD	Unconventional natural gas development
