# Supplementary material for: Greenness and Birth Outcomes in a Range of Pennsylvania Communities
Source: Int J Environ Res Public Health. 2016 Mar 11;13(3):311. doi: 10.3390/ijerph13030311 (PMC4808974; doi:10.3390/ijerph13030311)
Supplement: Supplementary file 1 [file ijerph-13-00311-s001.pdf]

Supplementary Materials: Greenness and Birth Outcomes in a Range of Pennsylvania Communities

Joan A. Casey, Peter James, Kara E. Rudolph, Chih-Da Wu and Brian S. Schwartz

Table S1. Unadjusted and adjusted associations of tertiles 2–3 *vs.* tertile 1 of residential greenness exposure and pregnancy outcomes in original population.

Variable [a]	Unadjusted [b]				Adjusted [b,c]			
	Term Birth Weight (g)	Small for Gestational Age Birth	Preterm Birth	5 min Apgar Score <7	Term Birth Weight (g)	Small for Gestational Age Birth	Preterm Birth	5 min Apgar Score <7
	β (95% CI)	OR (95% CI)	OR (95% CI)	OR (95% CI)	β (95% CI)	OR (95% CI)	OR (95% CI)	OR (95% CI)
**City, N**	2882	3278	3328	3364	2882	3278	3328	3364
Greenness T1	REF	1.0	1.0	1.0	REF	1.0	1.0	1.0
Greenness T2-3	31 (−1–62)	0.76 (0.61–0.95)	0.85 (0.68–1.06)	1.19 (0.85–1.68)	29 (−7–65)	0.75 (0.58–0.97)	0.82 (0.64–1.05)	1.15 (0.61–2.17)
**Borough, N**	4033	4514	4564	4620	4033	4514	4564	4620
Greenness T1	REF	1.0	1.0	1.0	REF	1.0	1.0	1.0
Greenness T2-3	10 (−15–36)	1.05 (0.86–1.27)	1.26 (1.01–1.56)	0.74 (1.01–1.39)	4 (−28–30)	1.06 (0.86–1.30)	1.19 (0.90–1.56)	0.94 (0.57–1.55)
**Township, N**	7326	8250	8343	8328	7326	8250	8343	8328
Greenness T1	REF	1.0	1.0	1.0	REF	1.0	1.0	1.0
Greenness T2-3	5 (−17–27)	0.90 (0.72–1.12)	0.98 (0.82–1.15)	1.23 (0.87–1.75)	2 (−21–24)	0.90 (0.72–1.12)	0.98 (0.82–1.15)	1.23 (0.87–1.75)

[a] Community-specific greenness (NDVI) tertile cutpoints: city = 0.43; borough = 0.49; and township = 0.54; [b] Models included doubly robust standard errors to account for babies nested in women and women nested in communities using the Stata ivreg2 and logit2 programs; [c] All models were adjusted for year and season of birth and sex of the neonate; maternal characteristics: age at delivery, race/ethnicity, primary care patient status, ever smoking, pre-pregnancy body mass index, parity, number of antibiotic orders during pregnancy, receipt of Medical Assistance, delivery hospital, drinking water source, distance to nearest major road, proximity to swine livestock operations, number of unconventional natural gas wells within 20 km; and community socioeconomic deprivation quartile and community walkability. The birth weight model was additionally adjusted for gestational age. Interactions that improved model fit were included.

Table S2. Unadjusted and adjusted associations of continuous residential greenness exposure and pregnancy outcomes in original and restricted populations.

Greenness, NDVI 1250 m	Unadjusted [a]				Adjusted [a,b]			
	Term Birth Weight (g)	Small for Gestational Age Birth	Preterm Birth	5 min Apgar Score <7	Term Birth Weight (g)	Small for Gestational Age Birth	Preterm Birth	5 min Apgar Score <7
	β (95% CI)	OR (95% CI)	OR (95% CI)	OR (95% CI)	β (95% CI)	OR (95% CI)	OR (95% CI)	OR (95% CI)
**Overall**								
Per 0.1 unit, ORIG.	16 (8–25)	0.94 (0.89–1.00)	1.01 (0.95–1.07)	1.03 (0.91–1.16)	7 (−3–17)	0.97 (0.91–1.04)	0.98 (0.90–1.06)	1.09 (0.91–1.30)
Per 0.1 unit, REST.	21 (10–31)	0.93 (0.87–1.00)	0.98 (0.91–1.05)	1.03 (0.89–1.18)	7 (−3–17)	0.96(0.89–1.03)	1.00 (0.92–1.09)	1.12 (0.93–1.32)
**City**								
Per 0.1 unit, ORIG.	16 (−7–39)	0.96 (0.85–1.09)	0.94 (0.82–1.07)	1.09 (0.79–1.50)	21 (−7–49)	0.97 (0.91–1.04)	0.84 (0.83–0.97)	1.01 (0.61–1.68)
Per 0.1 unit, REST.	16 (−14–47)	0.98 (0.79–1.17)	0.96 (0.82–1.10)	1.26 (0.82–1.93)	22 (−13–56)	1.02 (0.80–1.30)	0.86 (0.74–1.00)	1.17 (0.67–2.05)
**Borough**								
Per 0.1 unit, ORIG.	4 (−10–19)	1.05 (0.94–1.16)	1.12 (1.00–1.25)	0.82 (0.65–1.03)	4 (−13–21)	1.03 (0.90–1.17)	1.13 (0.98–1.27)	0.88 (0.65–1.17)
Per 0.1 unit, REST.	−4 (−19–11)	1.06 (0.95–1.17)	1.18 (1.03–1.36)	0.80 (0.60–1.07)	1 (−19–21)	1.02 (0.90–1.16)	1.14 (0.96–1.38)	0.91 (0.63–1.31)
**Township**								
Per 0.1 unit, ORIG.	−1 (−14–11)	1.00 (0.92–1.10)	1.03 (0.95–1.12)	1.11 (0.93–1.32)	−1 (−14–12)	1.04 (0.93–1.17)	1.05 (0.93–1.18)	1.20 (0.96–1.52)
Per 0.1 unit, REST.	1 (−13–14)	1.00 (0.90–1.11)	1.03 (0.93–1.13)	1.14 (0.92–1.41)	−1 (−15–13)	1.03 (0.90–1.17)	1.01 (0.89–1.15)	1.20 (0.92–1.58)

[a] Models included doubly robust standard errors to account for babies nested in women and women nested in communities using the Stata ivreg2 and logit2 programs. ORIG. = original population; REST. = restricted population; [b] All models were adjusted for year and season of birth and sex of the neonate; maternal characteristics: age at delivery, race/ethnicity, primary care patient status, ever smoking, pre-pregnancy body mass index, parity, number of antibiotic orders during pregnancy, receipt of Medical Assistance, delivery hospital, drinking water source, distance to nearest major road, proximity to swine livestock operations, number of unconventional natural gas wells within 20 km; and community socioeconomic deprivation quartile and community walkability. The birth weight model was additionally adjusted for gestational age. Interactions that improved model fit were included.

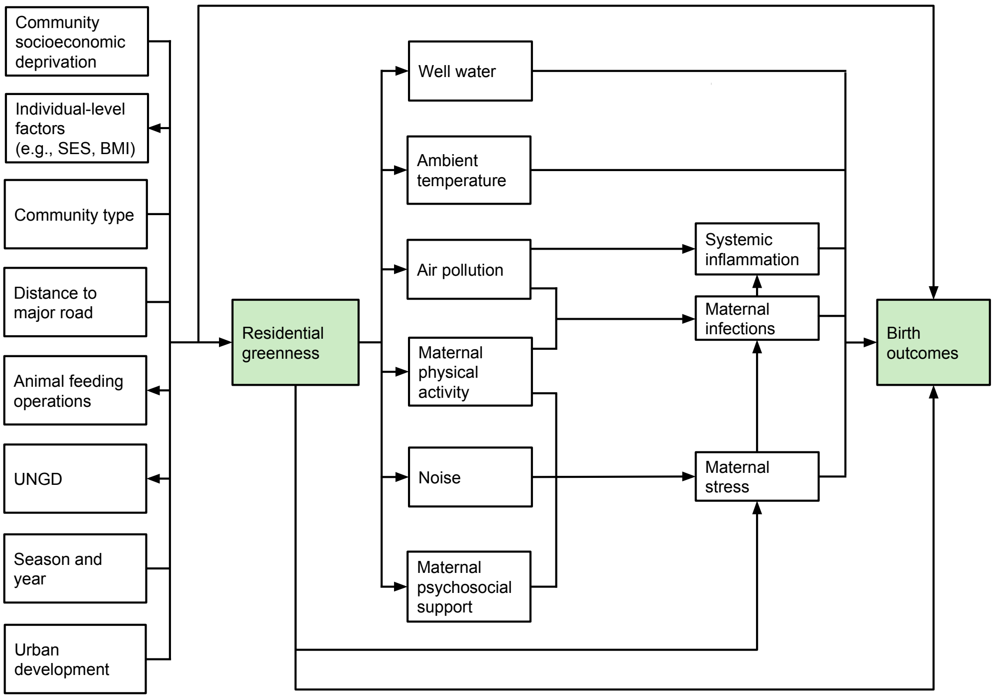

Figure S1. Conceptual framework with relations among residential greenness, confounders, mediators and birth outcomes. We hypothesized that community socioeconomic deprivation and urban development would primarily reduce residential greenness and that certain community types, seasons, and years, and greater distances to nearest major road would primarily increase residential greenness. We hypothesized that the relations between individual-level factors (e.g., socioeconomic status (SES), and pre-pregnancy body mass index (BMI)), animal feeding operations (AFOs), and unconventional natural gas development (UNGD) were likely to be bi-directional (*i.e.*, wealthier individuals may plant more trees, but greener neighborhoods might also attract individuals with more money; and greenness causes UNGD and AFOs because these are both primarily sited in rural and not built-up areas, but AFOs and UNGD also both remove vegetation thereby reducing greenness). We chose to treat these three exposures as confounders in our analysis because we believe that represents the primary causal direction. Each of the eight confounders can also cause poor birth outcomes. Residential greenness may directly lead to improved birth outcomes, however we hypothesized most of the effect was mediated by other factors. Residential greenness can reduce ambient temperatures during times of heat stress, improving birth outcomes. It can affect air pollution (e.g., reduce $PM_{2.5}$ and ozone, but increase volatile organic compounds) and increase maternal physical activity, both of which may reduce maternal infections and systemic inflammation, leading to improved birth outcomes. Greenness itself may reduce maternal stress and can also dampen noise and provide opportunities for increased maternal psychosocial support, which may in turn reduce maternal stress, again improving birth outcomes. Finally, mothers living in greener areas are more likely to rely on well water, which has been linked to poor birth outcomes (e.g., exposure to nitrates).

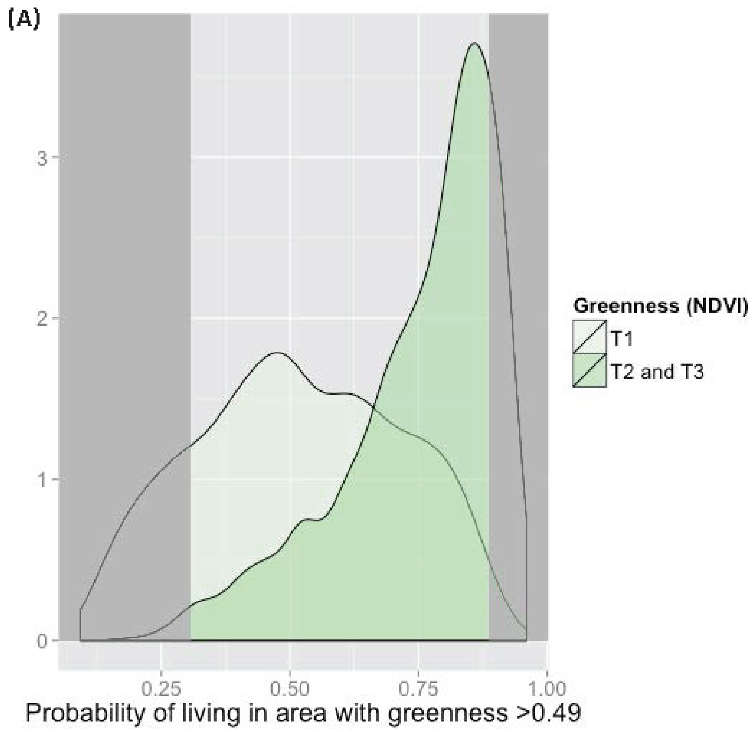

(**A**) Boroughs

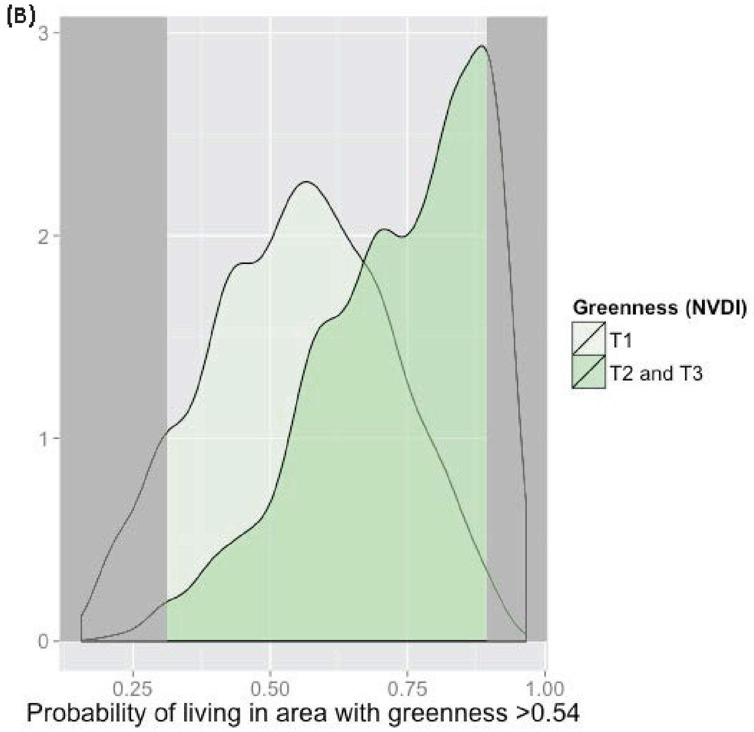

(**B**) Townships

Figure S2. *Cont.*

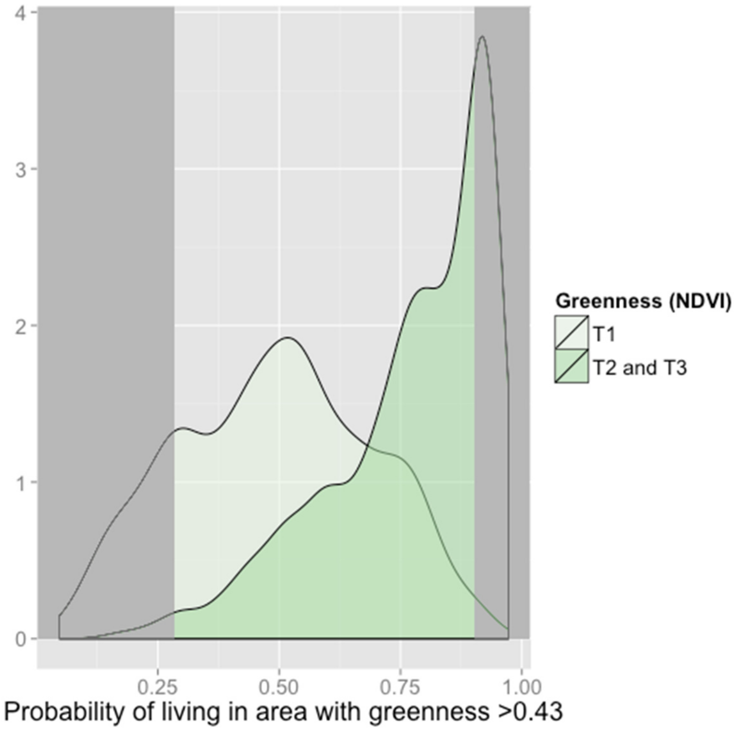

(**C**) Cities

Figure S2. Propensity score distributions for patients in (**A**) boroughs; (**B**) townships; and (**C**) cities comparing those with NDVI values in the 2nd and 3rd tertiles (darker green) to the 1st (lighter green). The propensity score is the predicted probability of living in tertiles 2 or 3 (NDVI > 0.49 and 0.54 for boroughs and townships, respectively), given baseline covariates. The analysis was restricted to participants with propensity scores outside of dark grey shaded regions to ensure that those living in green and non-green environments were comparable.

© 2016 by the authors; licensee MDPI, Basel, Switzerland. This article is an open access article distributed under the terms and conditions of the Creative Commons by Attribution (CC-BY) license (http://creativecommons.org/licenses/by/4.0/).
